# Validity and reliability of simple measurement device to assess the velocity of the barbell during squats

**DOI:** 10.1186/s13104-017-3012-z

**Published:** 2017-12-06

**Authors:** Silvio Lorenzetti, Thomas Lamparter, Fabian Lüthy

**Affiliations:** 1 Institute for Biomechanics, HCP H 21.1, ETH Zurich, Leopold-Ruzicka-Weg 4, 8093 Zurich, Switzerland; 2 Swiss Federal Institute of Sport Magglingen (SFISM), 2532 Magglingen, Switzerland

**Keywords:** Training monitoring, Linear position transducer (LPT), Strength exercises

## Abstract

**Objectives:**

The velocity of a barbell can provide important insights on the performance of athletes during strength training. The aim of this work was to assess the validity and reliably of four simple measurement devices that were compared to 3D motion capture measurements during squatting. Nine participants were assessed when performing 2 × 5 traditional squats with a weight of 70% of the 1 repetition maximum and ballistic squats with a weight of 25 kg. Simultaneously, data was recorded from three linear position transducers (T-FORCE, Tendo Power and GymAware), an accelerometer based system (Myotest) and a 3D motion capture system (Vicon) as the Gold Standard. Correlations between the simple measurement devices and 3D motion capture of the mean and the maximal velocity of the barbell, as well as the time to maximal velocity, were calculated.

**Results:**

The correlations during traditional squats were significant and very high (r = 0.932, 0.990, p < 0.01) and significant and moderate to high (r = 0.552, 0.860, p < 0.01). The Myotest could only be used during the ballistic squats and was less accurate. All the linear position transducers were able to assess squat performance, particularly during traditional squats and especially in terms of mean velocity and time to maximal velocity.

**Electronic supplementary material:**

The online version of this article (10.1186/s13104-017-3012-z) contains supplementary material, which is available to authorized users.

## Introduction

To enhance the performance of well-trained athletes, specific variables of strength training are often required [1]. For the lower limb, traditional squats are used to enhance maximal force, and ballistic squats are used to enhance rapid power generation [1–3]. The velocity of the bar during squats appears to be a valid parameter for monitoring strength training [4, 5].

It has been shown that based on the average velocity of the bar during the concentric phase at submaximal loads, the one repetition maximum (1RM) weight can be estimated [4, 6, 7]. The decrease of the bar velocity within a set can be related to the fatigue of the musculoskeletal system [8]. Therefore, recording the bar velocity allows coaches to monitor and steer the training as well as define a lower velocity limit as criteria for finishing a set [7]. Furthermore, real-time feedback regarding the bar velocity seems to have a positive effect on training [9].

Recently, many different measurement devices, including linear position transducers (LPT) and accelerometer based systems, have been developed to assess kinematic parameters during strength training [10, 11], and according to [11], the establishing the validity and reliability of these devices is crucial. To assess the validity/reliability of such devices, previous studies have used force plates [10, 12], video [13], motion capture systems [11] or no reference system [14–16]. However, the assessment of measurement devices during traditional and ballistic squats with motion capture as a reference system is missing.

Therefore, the aim of this work was to determine the validity and reliability in terms of average and maximal velocity as well as the time to maximal velocity of the bar as assessed by four simple measurement devices during traditional and ballistic squats.

## Main text

### Methods

Nine participants (age: 30.9 ± 5.9 years; height: 182 ± 6 cm; weight: 92.0 ± 8.7 kg; 1RM: 171 ± 20 kg; experience in strength training 9.7 ± 5.5 years; bob athletes and powerlifters) were analyzed. All subjects were informed of the nature of the study and signed informed consent. The ETH Ethics Committee in Zürich, Switzerland (EK 2014-N-50) approved this study.

**Fig. 1:**
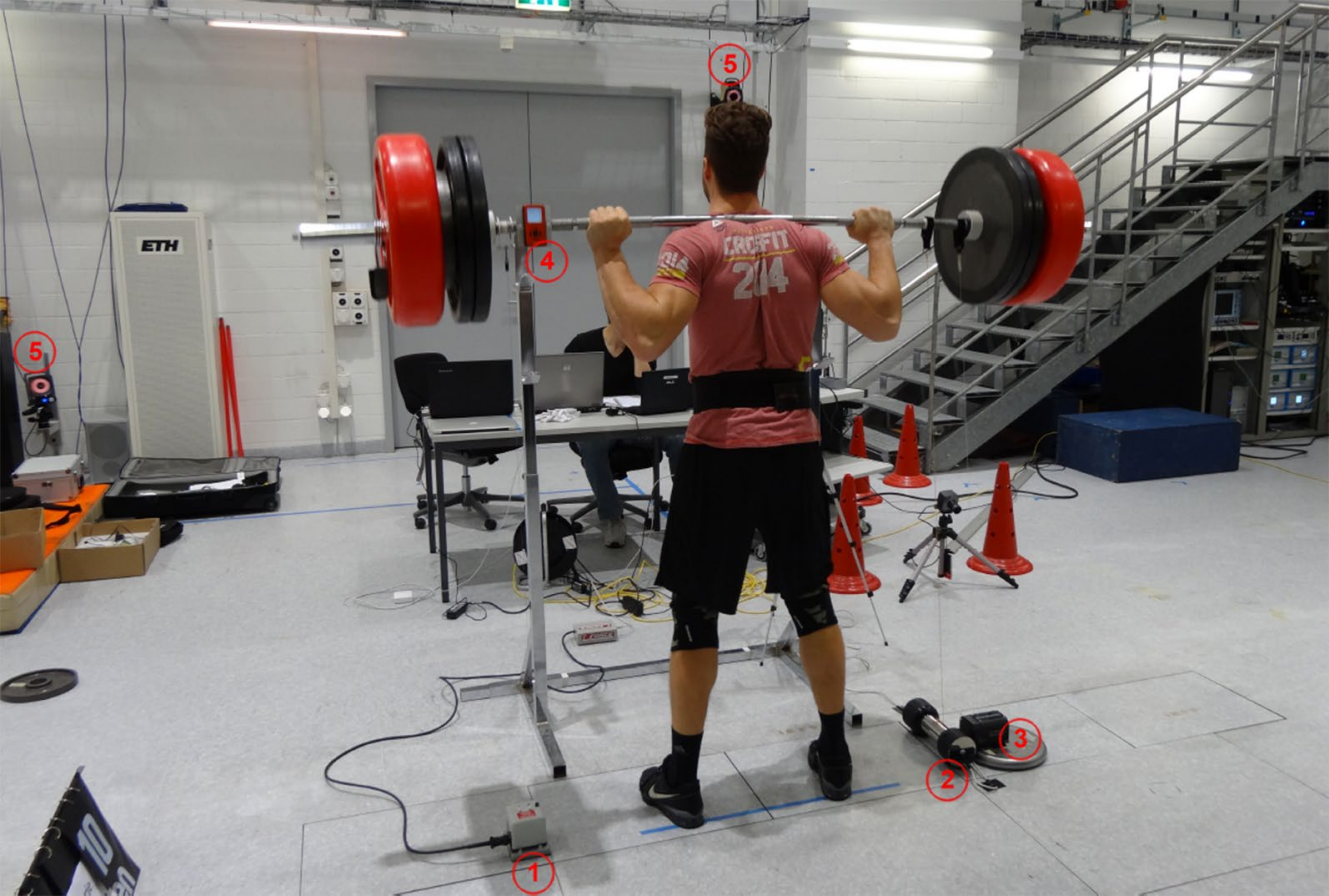
Simple measurement devices: 1 = T-force, 2 = Tendo unit, 3 = GymAware, 4 = Myotest, 5 = Vicon, the makers were placed at both ends of the bar

After a 5-min warm up, the participants performed 2 × 5 traditional squats (Fig. 1) with a weight of 70% of their 1RM and 2 × 5 ballistic squats (Additional file 1: Figure S1) with a weight of 25 kg, in a randomized order. The participants rested for 3 min between each set and for 5 min between the exercises.

Simultaneously, data was recorded from three linear position transducers [T-FORCE (version 2.3, T-FORCE Dynamic Measurement System, ERGOTECH Consulting, Murcia, Sp), Tendo Power (Tendo Power Analyzer Unit version 4.1.0, Tendo Sport Machines, Trecin, Slo) and GymAware (version 1.1.2, Kinetic Performance Technology, Mitchell, Aus)], an accelerometer-based system (Myotest, Myotest SA, Sion, CH), and a 3D motion capture system (16 camera Vicon System, Nexus 1.85, Vicon Motion Systems, Oxford, UK) with a sampling frequency of 100 Hz used as the gold standard (Fig. 1).

Based on the measurements from each device, the average velocity of the bar during the concentric phase (*V*_*mean*_), the maximum velocity of the bar during the concentric phase (*V*_*max*_), and the time to *V*_*max*_ (t *Vmax*) were calculated using Excel (version 14.5.2, Microsoft USA). In order to assess the validity and reliability, the root mean square error (RMSE), respectively the Pearson correlation between these measures obtained with the gold standard and the simple measurement devices were calculated. The device was reliable if the correlation has a value of r > 0.8 and a significant correlation coefficient is present [17]. All the statistical calculations were performed in SPSS (version 22, IBM, Chicago USA).

### Results

The resultant average velocity *V*_*mean*_ for the traditional and ballistic squats were 0.75 ± 0.13 and 1.56 ± 0.20 m/s, respectively. An evaluation of the data measured by the Myotest during the traditional squat was not directly possible.

#### Traditional squats

All the correlations to the gold standard were significant (Table 1). The RMSE for *V*_*max*_ was higher than the RMSE of *V*_*mean*_. For the average difference, no clear pattern was visible between the three different devices (Table 2).

**Table 1:** Differences, RMSE and correlations of the velocities of the bar *Vmean*, *Vmax* and *t* *Vmax* during traditional squats

	T-force	Tendo	GymAware
Δ *Vmean*			
Minimum	− 0.014	− 0.069	− 0.053
Maximum	0.136	0.137	0.158
Average	0.062	0.020	0.046
RMSE	0.070	0.046	0.064
*r Vmean*			
Correlation	0.970*	0.963*	0.958*
Δ *Vmax*			
Minimum	− 0.057	− 0.063	− 0.102
Maximum	0.369	0.422	0.388
Average	0.119	0.159	0.128
RMSE	0.151	0.194	0.163
*r Vmax*			
Correlation	0.933*	0.932*	0.957*
Δ *t Vmax*			
Minimum	− 0.075	− 0.090	− 0.006
Maximum	0.050	0.088	0.084
Average	0.010	0.031	0.037
RMSE	0.026	0.041	0.042
*r t Vmax*			
Correlation	0.985*	0.985*	0.990*

The units are m/s for the velocities and s for time

* Correlation is significant at a level of p < 0.01

**Table 2:** Differences, RMSE and correlations of the velocities of the bar *V*mean, *V*max and *t* *V*max during ballistic squats

	T-force	Tendo	GymAware	Myotest
Δ *Vmean*				
Minimum	− 0.265	− 0.521	− 0.460	− 0.739
Maximum	0.433	0.256	0.220	0.527
Average	0.102	− 0.083	− 0.091	0.149
RMSE	0.167	0.157	0.160	0.233
*r Vmean*				
Correlation	0.724*	0.770*	0.783*	0.610*
Δ *Vmax*				
Minimum	− 0.268	− 0.248	− 0.304	− 0.228
Maximum	0.694	0.787	0.795	1.124
Average	0.150	0.217	0.187	0.278
RMSE	0.263	0.315	0.304	0.418
*r Vmax*				
Correlation	0.810*	0.860*	0.852*	0.552*
Δ *t Vmax*				
Minimum	− 0.212	− 0.021	− 0.016	− 0.205
Maximum	0.221	0.285	0.237	0.040
Average	− 0.007	0.046	0.024	− 0.034
RMSE	0.045	0.064	0.046	0.054
*r t Vmax*				
Correlation	0.655*	0.604*	0.701*	0.700*

The units are m/s for the velocities and s for time

* Correlation is significant at a level of p < 0.01

#### Ballistic squats

All the correlations to the gold standard were significant (Table 1). The RMSE for *V*_*max*_ was higher than the RMSE of *V*_*mean*_. For the average difference, no clear pattern was visible between the three different LPT devices (Table 2). However, the velocity parameters were less reliable when measured using the accelerometer-based device.

### Discussion

In this study, the reliability and validity of four different devices used to assess two different bar velocities during traditional and ballistic squats were evaluated.

All three LPT devices were able to reliably measure the parameters *V*max, *V*mean and *t V*max. This is in agreement with results from the evaluation of the Tendo and T-Force systems with squats using a Smith machine and weight of 40 kg [14]. It is worth noting that the RMSE of *V*_*max*_ was larger than the other parameters. This indicates that *V*_*max*_ is a less robust measure and the values should be handled with great care.

A possible reason why the Myotest was not usable for evaluation of the traditional squats is the fact that here free squats were analyzed and not squats using a Smith machine. Resulting difficulties with the rotation of the bar have previously been reported [18]. No clear difference was observed between the three different LPT devices.

For the ballistic squats, the Myotest also produced reliable results. This is due to the fact that the Myotest procedure could be followed. Similar to our finding, Giroux et al. [10] observed higher correlations between the GymAware and a force plate compared to Myotest and a force plate. The observed correlations were likely lower when using the Myotest due to the fact that free squats were performed in the present study. While *tV*_*max*_ showed similar correlations for the two bar velocities, the *V*_*mean*_ and *V*_*max*_ showed less reliability at the higher velocity. In agreement with the findings of Jidovtseff [7], the parameters *V*_*mean*_ and *tV*_*mean*_ can be evaluated with the LPTs used here. This is important for the use of such devices in practice to allow the estimation of 1RMs and the fatigue during the workout.

The questions if a device is “valid” is clearly dependent on the research question, respectively on the magnitude of the values that should be quantified. In this study, the RMSE was < 11% for the three LPT for the traditional and ballistic squats and < 15% for the Myotest. Knowledge about the RMSE’s can provide evidence if a specific research question can be assessed using these devices.

Future research should focus either towards more valid and reliable devices for a cheaper prize or on the quantification during training in order to assess the volume, performance, fatigue, influence of different training settings in order to allow an individual training evaluation and steering. Maybe in the future it also might be possible to feed mechano-biological models for the adaptation process or use the data in order to prevent overload and overtraining.

To conclude, for the evaluation of squat performance at different squatting speeds, the three LPTs are reliable, and in particular, the parameters *V*_*mean*_ and *tV*_*max*_ can be used to monitor and guide workouts.

## Limitations

With the gold standard, the 3D path of the bar was evaluated; however, the LPT only analyzed linear motion. Therefore, the bar path and the velocity is underestimated by the LPTs. However, this represents a “real life” setting similar to practice. A possibility to get rid of this limitation would be to use a smith press to perform the squats.

### Additional file

**Additional file 1:Figure S1.** Start and end position of the ballistic squat exercise.
