# Supplementary figures and images for: Validity and reliability of simple measurement device to assess the velocity of the barbell during squats
Source: BMC Res Notes. 2017 Dec 6;10:707. doi: 10.1186/s13104-017-3012-z (PMC5719516; doi:10.1186/s13104-017-3012-z)

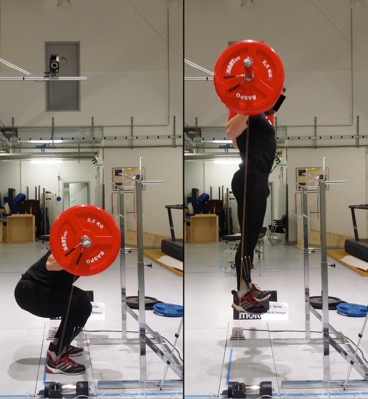

Supplement: Supplementary file 1 — Additional file 1:Figure S1. Start and end position of the ballistic squat exercise. [file 13104_2017_3012_MOESM1_ESM.jpg]
